# Structurally Dependent Self-Propulsion Behaviors of Pt-SiO_2_ Micromotors

**DOI:** 10.3390/nano16010073

**Published:** 2026-01-04

**Authors:** Le Zhou, Qian Zhao, Hongwen Zhang, Haoming Bao, Weiping Cai

**Affiliations:** 1 Key Lab of Materials Physics, Anhui Key Lab of Nanomaterials and Nanotechnology, Institute of Solid State Physics, Hefei Institutes of Physical Science, Chinese Academy of Sciences, Hefei 230031, China; zhoule@aqnu.edu.cn (L.Z.); zhaoqian@issp.ac.cn (Q.Z.); baohm@issp.ac.cn (H.B.); 2 Anhui Provincial Key Laboratory of Advanced Catalysis and Energy Materials, Anhui Ultra High Molecular Weight Polyethylene Fiber Engineering Research Center, School of Chemistry and Chemical Engineering, Anqing Normal University, Anqing 261433, China

**Keywords:** Pt-SiO_2_ micromotors, self-propulsion, structure-dependent motion, Pt-dragging/pushing motion, hydroxyl-mediated driving force

## Abstract

The structural dependence of self-propelled motion in micro/nanomotors is essential for effectively predicting and controlling their dynamic behaviors. In this study, platinum–silica (Pt-SiO_2_) micromotors, with structures ranging from spherical Janus to dimer configurations, are fabricated through conventional template-assisted deposition, followed by annealing. These structures are used to investigate how geometry influences motion. Our results demonstrate that the architecture of the Pt-SiO_2_ micromotor strongly affects its propulsion mode and trajectory in solution. When immersed in a hydrogen peroxide (H_2_O_2_) solution, spherical Janus Pt-SiO_2_ micromotors exhibit quasi-linear motion, driven by the Pt side (Pt pushing). In contrast, dimeric structures and intermediate forms varied from Janus to dimer display quasi-circular trajectories with continuously changing directions, characteristic of Pt-dragging motion. We reveal that these distinct propulsion behaviors stem from differences in the spatial distribution of Pt on the SiO_2_ sphere surface. Variations in Pt distribution alter the exposed silica surface area—rich in hydroxyl groups—which modulates the driving force and causes the resultant force acting on the micromotor to deviate from its mass center axis (or the axis connecting the mass centers of the Pt component and silica sphere), thereby inducing circular motion. This study offers a versatile strategy for fabricating Pt-SiO_2_ micromotors with tailored structures and advances the fundamental understanding of structure-dependent self-propulsion mechanisms.

## 1. Introduction

In recent years, self-propelled micro/nanomotors (or colloidal motors) have attracted significant attention due to their promising applications in intelligent systems across environmental [1,2,3,4,5], biomedical [6,7,8,9], and other fields [10,11]. These motors typically comprise two distinct components—a chemically active region and an inert one—or consist entirely of active materials, with an asymmetric structure. Their self-propulsion is driven by external energy sources, such as chemical energy derived from the decomposition of chemical fuels (e.g., H_2_O_2_) on the active component (e.g., Pt). The resulting motion behaviors are generally governed by various mechanisms, including bubble propulsion [12,13,14], self-electrophoresis [15,16], and self-diffusiophoresis [17,18,19,20], among others [21,22,23], depending on factors such as the motor’s intrinsic properties, size, and dynamic characteristics.

For better practical applications, it is crucial to control micro/nanomotors’ motion behaviors, such as their direction and speed, and the motor’s motion modes (e.g., active component-dragging or active component-pushing motion), which are closely associated with some internal (e.g., motor’s structure, shape, and composition distribution) and external (fuel concentration, environmental temperature, viscidity of fluid, etc.) factors. Until now, there have been many reports on the influences of external conditions on the motors’ motion behaviors. For instance, Gao et al. [24] prepared spherical Pd-Al Janus micromotors by coating Pd thin film on the surface of half Al microspheres, and found that these spherical Janus micromotors could exhibit different motion modes in different solutions [e.g., the active Pd-pushing type motion in H_2_O_2_ solution, and the Pd-dragging type motion in non-neutral (acidic or basic) solutions] due to the chemical reactions occurring at different areas on the Janus. Chen et al. [25] prepared spherical Pt/Au-TiO_2_ Janus micromotors and found that their motion mode could change from Pt-pushing type to Pt-dragging type in H_2_O_2_ solution, just via external light stimuli. Xiao et al. [26] reported that spherical Pt-TiO_2_ Janus micromotors could achieve significant speed increase in water under the light and alternative electric fields, where their speed was faster than the simple sum of the speeds induced only by either field. In short, these external factor-based works really broaden motors’ application scenarios.

Beyond external conditions, the intrinsic properties of motors—such as their structure, shape, and composition distribution—fundamentally govern their motion behaviors, propulsion efficiency, and potential applicability in specific environments. Among these internal factors, structure stands out as a key element influencing self-propulsion in fluid media. For instance, Ji et al. [27] synthesized spherical poly n-isopropylacrylamide (PNIPAM)@Au-Pt Janus bimetallic composite particles and observed that temperature-induced structural or conformational changes in the PNIPAM chains triggered a shift in the propulsion mechanism from self-electrophoresis to self-diffusiophoresis in H_2_O_2_ solution, illustrating how structural alterations can modulate the motion mechanism. In another study, Zhou et al. [28] fabricated Pt- and polymer-based micromotors with diverse structural features—including curved surfaces, flat planes, sharp edges, and pointed ends—and demonstrated that these structural variations led to differences in motion speed in H_2_O_2_ solution. Notably, Valadares et al. [29] reported that Pt-SiO_2_ dimer nanomotors exhibited Pt-dragging motion with quasi-linear and quasi-circular trajectories in H_2_O_2_, though the underlying origin of this behavior was not fully elucidated. While the influence of structure on motor speed is relatively straightforward to interpret, due to its direct effect on propulsion power and motion resistance, the shift in motion modes appears to involve deeper mechanistic aspects that remain debated and underexplored.

Here, we focus on structure-based motion manipulation in the fuel (H_2_O_2_)-contained solution for Pt-SiO_2_ micromotors, with structures varied from spherical Janus to dimer. These micromotors were obtained via template-assisted deposition, followed by annealing treatment. It has been found that the Pt-SiO_2_ micromotors exhibit structure-dependent self-propulsion behaviors in H_2_O_2_ solutions. Typically, the spherical Janus micromotors, built of Pt hemispherical shell-coated SiO_2_ microspheres, could exhibit quasi-directional (or linear) motion in a Pt shell-pushing way, whereas the Pt-SiO_2_ dimer micromotor (or the SiO_2_ microsphere attached with an irregularly shaped Pt particle) and the Pt-SiO_2_ micromotors with structures between spherical Janus and dimer (mostly, the SiO_2_ microsphere is attached with several Pt nanoparticles on its hemispherical surface) would exhibit Pt dragging and quasi-circular motion. The details are provided in the following sections.

## 2. Experimental Section

### 2.1. Materials and Chemicals

Monodisperse SiO_2_ microspheres (approximately 2 μm in diameter) were obtained from Changzhou Junyi New Materials Technology Co., Ltd. (Changzhou, China), and a Pt target (Φ50 × 1.0 mm) was acquired from Hefei Kejing Materials Technology Co., Ltd. (Hefei, China). All chemicals, including n-butanol and H_2_O_2_, were purchased from Sinopharm Chemical Reagent Co., Ltd. (Shanghai, China), and used as received, without further purification. Deionized water (resistivity of 18.2 MΩ·cm at 25 °C) was used throughout all experiments.

### 2.2. Preparation of Pt-SiO_2_ Micromotors with Various Structures

**Scheme 1:**
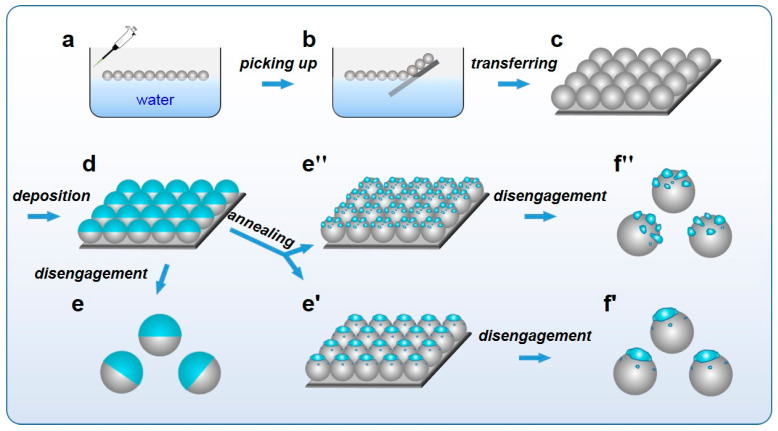
The schematic illustration of fabricating Pt-SiO_2_ micromotors with various structures. (**a**) Self-assembled SiO_2_ microsphere monolayer floating on water; (**b**) transfer of the monolayer onto a Si wafer; (**c**) the SiO_2_ monolayer on the Si wafer serving as template; (**d**) SiO_2_ monolayer after Pt deposition; (**e**) spherical Pt-SiO_2_ Janus microparticles obtained after release from the wafer; (**e**′,**e**″) SiO_2_ monolayer on the wafer after Pt deposition for 600s and 300s, respectively, followed by annealing at 900 °C for 180 min in air; (**f**′,**f**″) corresponding Pt-SiO_2_ particles after disengagement from the wafer.

The Pt-SiO_2_ micromotors with diverse structures were fabricated through a template-assisted deposition process, followed by annealing, as outlined in Scheme 1.

First, a SiO_2_ colloidal monolayer was prepared using a gas–liquid interfacial self-assembly method, following previously reported procedures [29,30,31]. Specifically, 0.1 g of monodisperse SiO_2_ microspheres was dispersed in 1 mL of n-butanol, via ultrasonic vibration, to form a colloidal suspension. Then, 0.2 mL of this suspension was slowly introduced along the inner wall of a beaker filled with water, using a pipette, to facilitate self-assembly. After standing for 1 h, a faint white self-assembled SiO_2_ monolayer formed and floated on the water surface (Scheme 1a). This monolayer was subsequently lifted using a Si wafer (2 cm × 2 cm) (Scheme 1b), transferred onto the wafer, and dried to serve as a template (Scheme 1c). Next, a thin Pt layer was deposited onto the SiO_2_ monolayer-covered wafer via ion sputtering, under a vacuum of 30 m Torr, and a sputtering current of 30 mA, corresponding to a deposition rate of 0.5 nm/s (Scheme 1d). By adjusting the deposition time and performing subsequent annealing treatments before release from the Si wafer, Pt-SiO_2_ micromotors with various architectures were obtained.

If Pt deposition was carried out for 300s, spherical Pt-SiO_2_ Janus particles were obtained after disengagement from Si wafer (Scheme 1e). The disengagement was achieved by immersing the Pt-deposited SiO_2_ monolayer on the Si wafer in water, and then sonicating at 400 W for 1 min. Alternatively, if the SiO_2_ monolayer-covered wafer was deposited with Pt for 600s and then annealed at 900 °C for 180 min in air, Pt-SiO_2_ dimers were fabricated (Scheme 1d,e′). Also, if the same procedures as those for dimers were used, but Pt deposition duration was 300s or less, we could obtain the various transition structures between the dimer and spherical Janus (Scheme 1d,e″). Finally, the monodispersed Pt-SiO_2_ micromotors with various structures were obtained by detaching the Pt-SiO_2_ particles from Si wafer. This was also achieved by immersing the annealed Pt-SiO_2_-covered wafer in water, followed by 1 min. of sonication (Scheme 1f′,f″).

### 2.3. Characterizations and Motion Observations

The morphology of the samples was characterized using a field emission scanning electron microscope [FESEM, Sirion 200 (FEI, Eindhoven, The Netherlands)) equipped with an energy-dispersive spectrometer (EDS). An X’Pert Phillips diffractometer (PANalytical, Almelo, The Netherlands) was used to acquire the X-ray diffraction (XRD) patterns.

To observe the motion of the Pt-SiO_2_ micromotors, aqueous H_2_O_2_ solutions, with concentrations ranging from 0 to 10 wt%, were used. A droplet of the micromotor suspension in H_2_O_2_ solution was placed into a slide groove, covered with a coverslip, and sealed. The motion was recorded under an optical microscope (Leica DM4B).

The recorded video data, containing the actual motion trajectories, were processed using the software Premiere Pro (version 14.0) to generate continuous image sequences. The resulting images were analyzed with the image processing software Image J (version 1.52g) to extract pixel-based displacement and positional data. Actual motion parameters were determined by calibrating the scale bar with the corresponding pixel values.

## 3. Results and Discussion

### 3.1. Morphology and Structure of Micromotors

**Figure 1:**
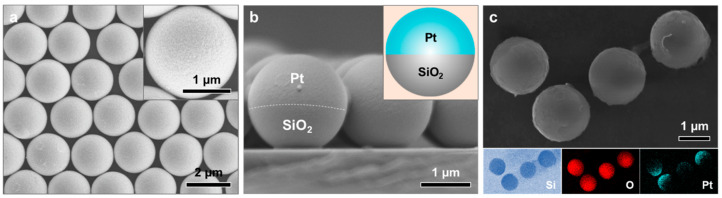
FESEM images of the sample after 300 s of Pt sputter deposition on the SiO_2_ monolayer-covered Si wafer: (**a**) top-view and (**b**) cross-sectional view. The insets in (**a**,**b**) show a magnified image and a schematic illustration, respectively, of an individual SiO_2_ microsphere coated with a hemispherical Pt shell. (**c**) Hemispherical Pt shell-coated SiO_2_ microspheres released from the Si wafer, along with the corresponding EDS elemental mappings.

A monolayer template of 2 μm SiO_2_ microspheres was fabricated on a Si wafer using gas–liquid interfacial self-assembly, followed by transfer, as shown in Figure S1. Subsequent sputter deposition of Pt onto the monolayer resulted in the formation of a thin Pt layer on the upper surfaces of the SiO_2_ microspheres. Subsequent sputter deposition of Pt onto the monolayer resulted in the formation of a thin Pt layer on the upper surfaces of the SiO_2_ microspheres. Figure 1a,b presents the sample after 300 s of Pt deposition, corresponding to a thickness of approximately 150 nm. As shown, each SiO_2_ microsphere is uniformly coated with a continuous Pt hemispherical shell. These spherical Pt–SiO_2_ Janus composite particles, referred to as Janus micromotors, were then released from the Si substrate, as schematically illustrated in Figure 1c.

**Figure 2:**
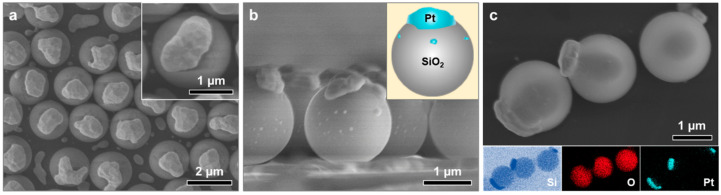
FESEM images of 300 nm Pt-coated SiO_2_ microspheres after annealing: (**a**) top view and (**b**) cross-sectional view of the microspheres on a Si wafer. The insets in (**a**,**b**) present a magnified image and a schematic illustration, respectively, of an individual dimer structure. (**c**) Pt-coated SiO_2_ microspheres disengaged from the Si wafer along with their corresponding EDS elemental mappings.

If the Pt coating on SiO_2_ monolayer was much thicker than 150 nm, the subsequent annealing at a high temperature (900 °C) would form the Pt-SiO_2_ dimers on the Si wafer, as shown in Figure 2a,b, corresponding to 300 nm of Pt deposition thickness. Each SiO_2_ microsphere is attached with one irregularly shaped Pt particle with about 1 µm in planar size, in addition to the few sporadic tiny Pt nanoparticles, showing dimer structure. Finally, the Pt-SiO_2_ dimer micromotors could be obtained by ultrasonic disengagement, as shown in Figure 2c. The Pt particles were not oxidized even after annealing, which could be well-supported by XRD pattern shown in Figure S2.

**Figure 3:**
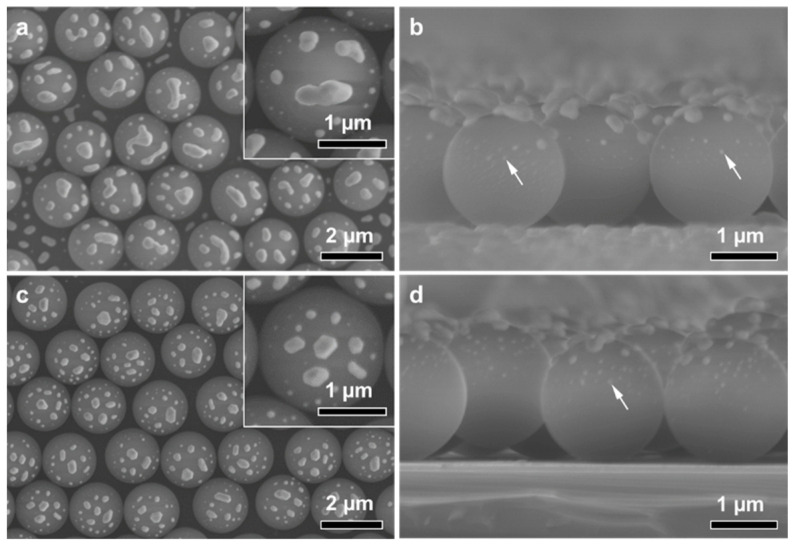
FESEM images of Pt-coated SiO_2_ microspheres after annealing for Pt deposition thicknesses of 150 nm (**a**,**b**) and 90 nm (**c**,**d**). (**a**,**c**) Top views (insets: local magnifications); (**b**,**d**) cross-sectional views and the arrows point to the tiny Pt particles.

Furthermore, if the Pt coating on monolayer SiO_2_ microspheres was not thick enough (that is, 150 nm or less), the subsequent annealing would lead to Pt-SiO_2_ composite particles with the transition structures between spherical Janus and dimer, as shown in Figure 3. When the Pt deposition thickness was about 150 nm, the integrated hemispherical Pt shell on a SiO_2_ microsphere was transformed into several isolated, irregularly shaped Pt particles, ~100–200 nm in size, after annealing (The sample is denoted as Pt-SiO_2_-150). These isolated Pt particles were decorated on the hemispherical surface of the SiO_2_ microsphere, as shown in Figure 3a,b. Also, if the Pt deposition layer was thinner (that is, ~90 nm), the final Pt-SiO_2_ micromotors (or Pt-SiO_2_-90, in short) were similar in the structure or conformation to Pt-SiO_2_-150, except that the isolated, irregularly shaped Pt particles were smaller (~50–150 nm) in size (Figure 3c,d). For both samples, there inevitably exist some sporadic tiny Pt particles, less than ~30 nm in size, on the SiO_2_ microspheres, in addition to the bigger isolated Pt particles, as indicated by arrows in Figure 3b,d.

### 3.2. Surface/Interface Tension-Induced Pt Shell’s Shrinking Mechanism

**Figure 4:**
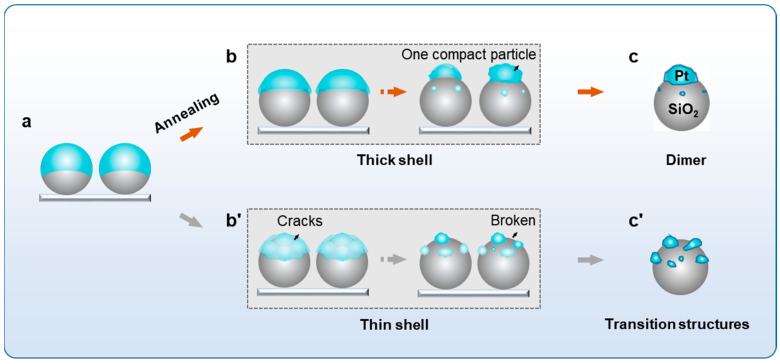
Schematic illustration of surface/interface tension-induced Pt shell’s shrinking mechanism for the Pt-SiO_2_ composite particles with various structures. (**a**) Spherical Pt-SiO_2_ Janus particle. (**b**,**b**′) annealing-induced Pt shell shrinking. (**c**,**c**′) Final Pt-SiO_2_ particles, dimer and transitional structures between Janus and dimer, respectively.

The above annealing-induced conformational change in Pt-SiO_2_ particles is easily understood. It could be attributed to the surface/interface tension-induced shrinking of the Pt shell along the surface of SiO_2_ microspheres, as illustrated in Figure 4. Briefly, due to surface/interface tension, the Pt hemispherical shell would de-wet or shrink along the surface of SiO_2_ microspheres during annealing at a high temperature. When the deposition thickness was large enough (that is, 300 nm), the Pt shell would be strong enough to avoid cracking during annealing and, hence, form an irregularly shaped and compact Pt particle on each SiO_2_ sphere (Figure 4b) or dimer (Figure 2 or Figure 4c). On the contrary, if the deposition thickness was small (that is, 150 nm or less), the Pt shell would be weak and easily broken due to surface/interface tension-induced shrinking during annealing (Figure 4b′). In this case, several smaller Pt particles would be formed on each SiO_2_ microsphere after annealing (Figure 3 or Figure 4c′).

### 3.3. Structurally Dependent Self-Propelled Behaviors

The self-propelled behaviors in H_2_O_2_ solutions were examined for the Pt-SiO_2_ composite particles with different conformations, which exhibit significant structural dependence.

#### 3.3.1. Pt-SiO_2_ Janus Micromotors

**Figure 5:**
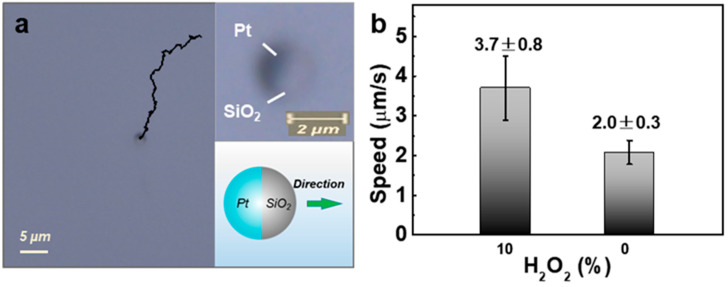
Self-propelled motion behavior of the Pt-SiO_2_ Janus micromotors in 10 wt% H_2_O_2_ solution. (**a**) A typical motion trajectory (from Video S1). Top right inset: an enlarged view of a spherical Janus particle, consisting of a dark Pt shell and a gray SiO_2_ hemisphere; bottom-right inset: schematic of the Pt-SiO_2_ Janus micromotor showing the Pt shell-pushing propulsion direction (or the arrow points to). (**b**) Histogram of the average linear speed of the Pt-SiO_2_ Janus micromotors in H_2_O_2_-containing and H_2_O_2_-free aqueous solutions.

When immersed in H_2_O_2_ solution, Pt-SiO_2_ Janus micromotors show a directional motion with quasi-linear trajectory, as shown in Video S1 and Figure 5a. This is attributed to the catalytic decomposition of H_2_O_2_ by the active Pt hemispherical shell, and in agreement with the previous reports [29,32]. These Pt-SiO_2_ Janus micromotors move in a Pt shell-pushing way (insets in Figure 5a). In contrast, these Janus micromotors in water, without H_2_O_2_, were localized within a small space and showed overlapping and irregular trajectories (Video S2 and Figure S3), typical Brownian movement. Furthermore, the average speed of the Janus micromotors was about 3.7 μm/s in 10 wt% H_2_O_2_ solution, in contrast to the ~2.0 μm/s speed of Brownian motion in water (Figure 5b).

#### 3.3.2. Pt-SiO_2_ Dimer Micromotors

**Figure 6:**
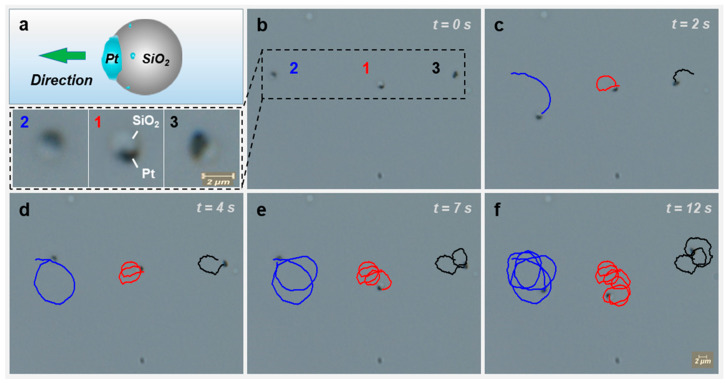
Self-propelled motion behavior of Pt-SiO_2_ dimer micromotors in 10 wt% H_2_O_2_ aqueous solution. (**a**) Schematic illustration of a Pt-SiO_2_ dimer micromotor with Pt-dragging motion orientation. (**b**–**f**) Time-lapse images (from Video S3) showing the motion trajectories of three individual dimer micromotors (labeled as No. 1, 2, and 3) in H_2_O_2_ solution. The inset in dotted box below (**a**) is an enlarged view of these three micromotors shown in (**b**–**f**).

Pt-SiO_2_ dimer micromotors move in a different mode from Pt-SiO_2_ Janus micromotors in H_2_O_2_ solution. Figure 6 shows the motion behaviors for the Pt-SiO_2_ dimers in 10 wt% H_2_O_2_ solution. They move in a Pt-dragging way, as illustrated in Video S3 and Figure 6a. The micromotor orientation during motion for the Pt-SiO_2_ dimers is opposite to that of the Pt-SiO_2_ Janus micromotors, shown in Figure 5a. Figure 6b–f gives the time-lapse pictures captured from the motion video (Video S3), and the corresponding motion trajectories, for three Pt-SiO_2_ dimer micromotors with different-shaped Pt particles (marked as No. 1, 2, 3). Although, the Pt loading amounts of these three motors are slightly different, they were all moving in a Pt-dragging way and exhibiting quasi-circular motion trajectories, completely different from the above Pt-SiO_2_ Janus micromotors.

**Figure 7:**
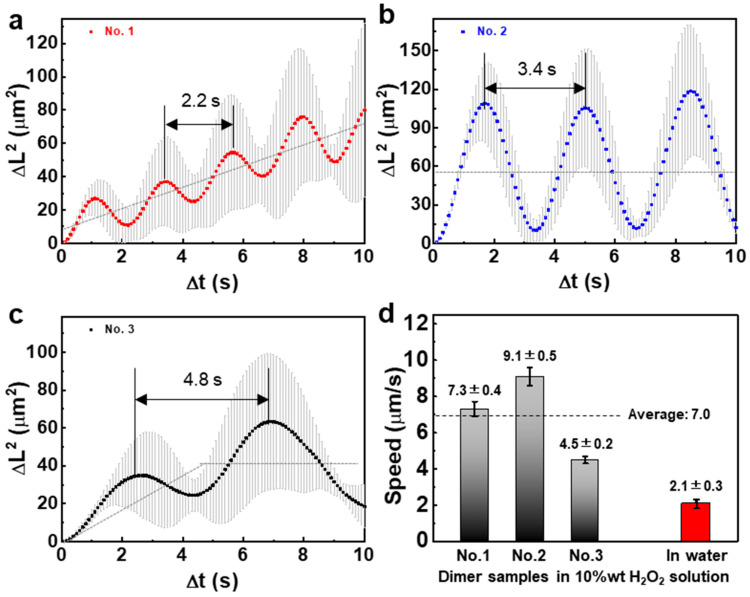
MSD curves of the Pt-SiO_2_ dimer micromotors in 10 wt% H_2_O_2_ aqueous solution, (**a**–**c**) corresponding to dimer micromotor Nos. 1, 2, 3 in Figure 6, respectively (The gray vertical lines denote error bars). (**d**) The histogram of line speeds for the three dimer micromotors in aqueous solution with or without 10 wt% H_2_O_2_. (The data were from Figure 6 and Figure S4).

Furthermore, from the motion trajectories shown in Figure 6, the curves of mean square displacement (MSD) were obtained for these three dimer micromotors, as illustrated in Figure 7a–c. These curves are all quasi-periodic oscillatory, which reflect some motion characteristic-related information. For micromotor No. 1, its MSD is oscillatory around a straight line, deviating from X-axis with motion time (Figure 7a). This indicates dual motion: the quasi-circular motion with the changing motion-center, or the superposition of the circular and directional motion. For micromotor No. 2, the MSD shows periodic oscillation, with a bigger amplitude, around a horizontal line (Figure 7b), indicating circular motion with a bigger motion radius and nearly fixed motion center during the observation. As for dimer micromotor No. 3, its MSD is similar to that of dimer No. 1 first, and then to that of dimer No. 2 (Figure 7c), indicating that its circular motion center shifts at the beginning, and then tends to be fixed. In addition, the periods of quasi-circular motion were estimated to be 2.2 s, 3.4 s, and 4.8 s for dimer micromotor Nos. 1–3, respectively. The difference in the oscillatory MSD curves (including circular motion period) among the three dimer micromotors should result from the shape or morphology of the Pt particle on a SiO_2_ sphere.

Also, the motion speeds were calculated for dimer micromotor Nos. 1–3, according to their motion trajectories and spent time, which were about 7.3, 9.1, and 4.5 μm/s, respectively, as given in Figure 7d. In addition, the micromotors with high speed (e.g., micromotor No. 2) tend to exhibit quasi-circular motion with big radius. Finally, the average speed of the three dimer micromotors in 10 wt% H_2_O_2_ solution is about 7.0 µm/s, which is much higher than that (3.7 µm/s) of the above Pt-SiO_2_ Janus motors. As for the dimer motors in water, they also show Brownian movement in a small space, with a speed of about 2.1 µm/s, as shown in Video S4 and Figure S4 and Figure 7d.

**Figure 8:**
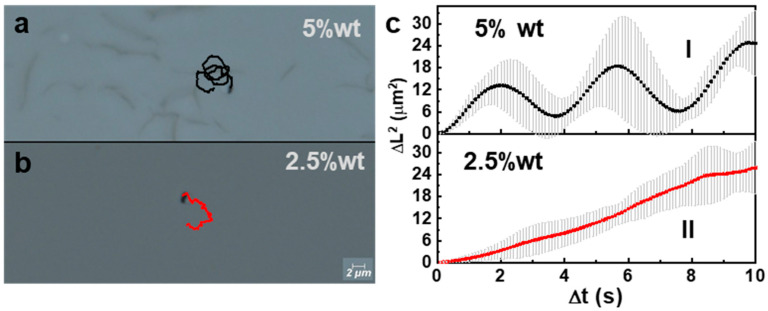
Motion behaviors of the dimer micromotors in aqueous solutions with low H_2_O_2_ concentrations over 12 s. (**a**,**b**) Complete motion trajectories in 5 wt% and 2.5 wt% H_2_O_2_ solutions, respectively. (**c**) The corresponding MSD curves (The gray vertical lines denote error bars).

Finally, the motion behavior of the dimer micromotors was examined in aqueous solutions with significantly lower H_2_O_2_ concentrations. In 5 wt% H_2_O_2_ solution, the dimers continued to exhibit Pt-dragging motion, albeit at a considerably reduced speed of 3.5 ± 0.5 μm/s compared to that in 10 wt% H_2_O_2_. Their trajectories remained quasi-circular, and the corresponding MSD curve displayed quasi-periodic oscillations, as shown in Video S5, Figure 8a, and curve I in Figure 8c. When the H_2_O_2_ concentration was further reduced to 2.5 wt%, the dimers showed only faint quasi-circular motion with very low speeds of 2.2 ± 0.3 μm/s (Figure 8b, curve II in Figure 8c), and an MSD profile that was nearly linear, which was similar to that of the Janus micromotors. Overall, the motion trajectories of the dimer micromotors exhibit a clear dependence on propulsion speed, which, in turn, correlates with H_2_O_2_ concentration: higher speeds or concentrations promote more pronounced circular motion.

#### 3.3.3. Pt-SiO_2_ Micromotors with Various Transition Structures

**Figure 9:**
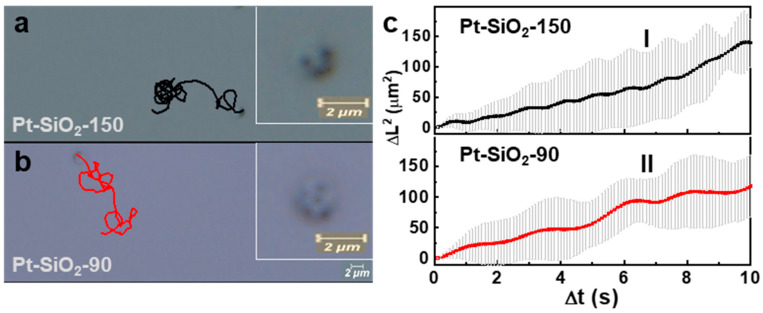
Motion behavior of the Pt-SiO_2_ micromotors, with transition structures between dimer and spherical Janus, in 10 wt% H_2_O_2_ solution within 12 s. (**a**,**b**) The complete motion trajectories for the Pt-SiO_2_-150 and Pt-SiO_2_-90 micromotors in the aqueous solution, respectively (data from Video S6). Insets: typical optical micrographs of the respective micromotors. (**c**) MSD curves for (I) Pt-SiO_2_-150 and (II) Pt-SiO_2_-90 (The gray vertical lines denote error bars).

Finally, the motion behaviors of the Pt-SiO_2_ micromotors with various transition structures between dimer and spherical Janus were examined. When a Pt-SiO_2_-150 particle, shown in Figure 3a,b, was immersed in 10 wt% H_2_O_2_ aqueous solution, it still exhibited quasi-circular motion in a Pt-dragging way, as illustrated in Figure 9a or Video S6a. In contrast to the dimers, the corresponding MSD curve, while still oscillatory, displays a much-reduced amplitude (curve I, Figure 9c; cf. Figure 7). The motion speed was as high as 8.0 µm/s. As for the Pt-SiO_2_-90 composite particles, with much lower Pt loading (Figure 3c,d), they also have the similar motion behaviors to the Pt-SiO_2_-150 micromotors, but with lower speed (6.3 µm/s), as shown in Video S6b and Figure 9b and curve II in Figure 9c).

In summary, when the Pt-SiO_2_ micromotors’ structures transit from spherical Janus to dimer, their motion modes change, from the Pt-pushing to Pt-dragging type, and the exhibited trajectory transitions from quasi-linear to quasi-circular, especially in an aqueous solution with high H_2_O_2_ concentration. These micromotors’ motions could have some potential applications. For instance, the quasi-linear motion for the spherical Pt-SiO_2_ Janus particles enables directional transport for targeted delivery in biomedicine; the quasi-circular motion or the quasi-circular motion with the changing motion-center for the Pt-SiO_2_ dimers, or the Pt-SiO_2_ micromotors with transition structures between dimer and spherical Janus, could provide the localized fluid-mixing useful in environmental catalysis or sensing.

### 3.4. Pt Distribution-Induced Motion Mode Variation

Now, let us discuss the conformationally dependent motion behaviors for *P*t-SiO_2_ micromotors. As previously reported [32,33], *P*t-SiO_2_ spherical Janus motors usually achieved self-propulsion via diffusiophoresis. Generally, when immersed in an H_2_O_2_ aqueous solution, the *P*t component of the micromotor acts as a catalyst for H_2_O_2_ decomposition, triggering the following reaction:

(1) $$2H_{2}O_{2}\overset{Pt}{\to }2H_{2}O+O_{2}$$

**Figure 10:**
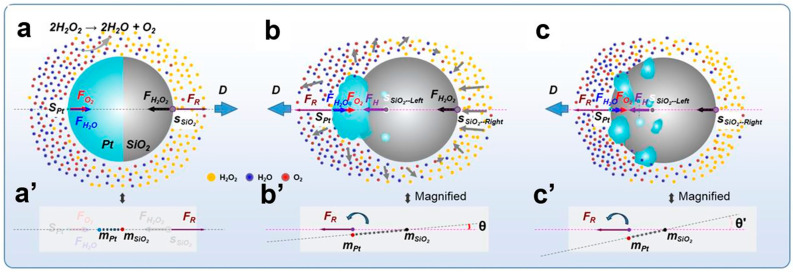
Schematic illustrations of motion orientation mechanism for Pt-SiO_2_ micromotors with different structures. (**a**) Spherical Janus. (**b**) Dimer. (**c**) Structure between spherical Janus and dimer. (**a**′–**c**′) are the illustrations of the resultant force-axis and the mass center-axis for the corresponding micromotors. D represents motion direction.

Surplus H_2_O and O_2_ molecules are produced, and their concentration gradients are formed on the side of *P*t component. On one hand, the repulsive forces between O_2_ and Pt (${\vec{F}}_{O_{2}}$), as well as H_2_O and *P*t (${\vec{F}}_{H_{2}O}$), would be generated due to the gradient-induced diffusion of H_2_O and O_2_, which acts on Pt component; on the other hand, the exposed surface of the SiO_2_ sphere in a micromotor would be struck by adjacent H_2_O_2_ molecules, resulting in a net force ${\vec{F}}_{H_{2}O_{2}}$ acting upon it, as shown in Figure 10. Consequently, the motion behaviors for *P*t-SiO_2_ micromotors arise from the resultant force ${\vec{F}}_{R}$ of the aforementioned three forces, or the following occurs:

(2) $${\vec{F}}_{R}\;=\;({\vec{F}}_{H_{2}O}+\;{\vec{F}}_{O_{2}})\;+\;{\vec{F}}_{H_{2}O_{2}}$$

Here, a force directed to the right is defined as positive, and negative if to the left. Obviously, when ${\vec{F}}_{R}$ > 0 or (${\vec{F}}_{H_{2}O}$ + ${\vec{F}}_{O_{2}}$) > ${\vec{F}}_{H_{2}O_{2}}$, the micromotors would move in a pushing way. Otherwise, if ${\vec{F}}_{R}$ < 0, the *P*t-dragging type motion would be seen. Also, if the resultant force ${\vec{F}}_{R}$ < 0 and is unparallel to the micromotor’s mass-center axis—the line between the two mass centers of *P*t component and silica sphere, forming an angle θ with it, as illustrated in Figure 10b′,c′—the micromotor would move with continuously changing directions, showing quasi-circular trajectories.

For a spherical Pt-SiO_2_ Janus micromotor, when immersed in H_2_O_2_ solution, the forces ${\vec{F}}_{H_{2}O}$ and ${\vec{F}}_{O_{2}}$ would be generated and act on the hemispherical Pt shell; meanwhile ${\vec{F}}_{H_{2}O_{2}}$ is formed and acts on the exposed silica surface (Figure 10a). Here, the forces ${\vec{F}}_{H_{2}O}$ and ${\vec{F}}_{O_{2}}$, as well as ${\vec{F}}_{H_{2}O_{2}}$, are all parallel to the mass-center axis of the Janus micromotor, or θ = 0°, due to its symmetry (Figure 10a′). Also, ${\vec{F}}_{H_{2}O}$ and ${\vec{F}}_{H_{2}O_{2}}$ could offset each other due to the nearly same amounts of produced H_2_O and unconsumed H_2_O_2_ on both sides of the Janus motor (see Equation (1)). Consequently, the sum (${\vec{F}}_{H_{2}O}$ + ${\vec{F}}_{O_{2}}$) would be greater than the force ${\vec{F}}_{H_{2}O_{2}}$, or ${\vec{F}}_{R}$ > 0, and the Janus motor moved in a Pt shell-pushing way, exhibiting an approximately directional motion trajectory (Video S1 and Figure 5), as previously reported [32,33].

For the Pt-SiO_2_ dimer micromotors, conformation is different from the spherical Janus structure. The Pt particle on SiO_2_ sphere was irregularly shaped, with much less exposed SiO_2_ surface area than that of a hemi-sphere. In this case, the H_2_O and O_2_ molecules generated by platinum catalysis also create a high concentration gradient near the exposed silica surface on the same side. Hydrogen bonding of additional H_2_O molecules, with hydroxyl groups on the exposed silica surface, gives rise to an additional force, ${\vec{F}}_{H}$ (a dragging force on the silica sphere), marked in Figure 10b. So, when a dimer was immersed in H_2_O_2_ solution, in addition to the forces ${\vec{F}}_{H_{2}O}$ and ${\vec{F}}_{O_{2}}$ acting upon the Pt particle at one end of the dimer, as well as the force ${\vec{F}}_{H_{2}O_{2}}$ on the exposed SiO_2_ hemisphere at the other end, a new force ${\vec{F}}_{H}$, with the same direction as ${\vec{F}}_{H_{2}O_{2}}$, would be generated, acting on the exposed silica surface on the platinum-bearing side of the dimer, or the following:

(3) $${\vec{F}}_{R}=\;({\vec{F}}_{H_{2}O}+\;{\vec{F}}_{O_{2}})\;+\;{\vec{F}}_{H}+\;{\vec{F}}_{H_{2}O_{2}}$$

Obviously, the force (${\vec{F}}_{H_{2}O}$ + ${\vec{F}}_{O_{2}}$) for the dimer was much smaller, due to much lower surface area of the Pt particle attached on SiO_2_ sphere, compared with the Janus micromotor. Considering the additional force ${\vec{F}}_{H}$, it is reasonable that (${\vec{F}}_{H_{2}O}$ + ${\vec{F}}_{O_{2}}$) < (${\vec{F}}_{H}$ + ${\vec{F}}_{H_{2}O_{2}}$), or ${\vec{F}}_{R}$ < 0. This results in the motion of the dimer in a Pt-dragging way. Additionally, due to the irregular shape of Pt particles, the resultant force ${\vec{F}}_{R}$, acting on the dimer motor, is no longer parallel to its mass-central axis, or θ > 0° (as shown in Figure 10b′). Under this resultant force, the dimer undergoes an approximately circular motion, in a Pt-dragging way, with the radius of this motion depending on the θ value, as shown in Figure 6 and Figure 8 and Video S3. As for the differences among dimers Nos. 1, 2, and 3 in motion speed and trajectories (or MSD curves) (Figure 6 and Figure 7), they should be attributed to the differences in shape, surface area, etc., of the Pt particles in these dimers.

As for the Pt-SiO_2_ motors with transition structures between Janus and dimer (e.g., Pt-SiO_2_-150 or Pt-SiO_2_-90), they also had exposed SiO_2_ surfaces on the Pt-bearing SiO_2_ hemisphere. Consequently, they were similar to the dimer motors, exhibiting motion in a Pt-dragging way and approximately circular trajectories in the H_2_O_2_ solution, due to the hydrogen bond effect, as shown in Figure 9 and Video S6.

Furthermore, to confirm the hydrogen bond effect, the exposed areas of the SiO_2_ spheres in dimers were modified with 1H,1H,2H,2H-perfluorodecyltrimethoxysilane [34,35], resulting in hydroxyl-free surfaces (details are provided in the Experimental Section of the Supporting Information). It has been shown that, in 10 wt% H_2_O_2_ solution, this hydroxyl-free Pt-SiO_2_ dimer exhibited a Pt-pushing motion at an average line speed of 3.0 µm/s, rather than a Pt-dragging mode, as demonstrated in Video S7 and Figure S5. This confirms the existence of hydroxyl group-induced additional force *F*_H_, which was large enough to change the dimer motion type. Further work is still needed for the mechanism of the structurally-dependent motion modes of micro/nanomotors.

## 4. Conclusions

In summary, this work presents a systematic investigation into the effect of microstructure on the self-propulsion of Pt-SiO_2_ micromotors. We have successfully fabricated a series of Pt-SiO_2_ micromotors, with structures ranging from spherical Janus to dimer, through a versatile template-assisted deposition and annealing method. The key finding is that these structural variations directly dictate the motors’ motion modes, as follows: spherical Janus motors exhibit Pt-pushing quasi-linear trajectories, whereas dimer and transitional structures demonstrate Pt-dragging quasi-circular motion. This behavioral dichotomy fundamentally stems from the distribution of the Pt catalyst on the SiO_2_ sphere surface, which modulates the exposed hydroxyl-rich silica area. This alteration in surface chemistry not only changes the driving force, but, crucially, causes a deviation in the resultant force from the mass central axis, thereby inducing rotational motion. Consequently, this study provides not only a flexible synthetic pathway for engineering micromotor architectures, but also delivers a deeper mechanistic understanding of how structure governs self-propulsion, paving the way for the rational design of micromotors with tailored motion behaviors for future applications.

## Data Availability

The original contributions presented in this study are included in the article. Further inquiries can be directed to the corresponding authors.

## Supplementary Materials

The following supporting information can be downloaded at: https://www.mdpi.com/article/10.3390/nano16010073/s1, Figure S1: FESEM image of the obtained SiO_2_ microsphere monolayer template; Figure S2: XRD patterns of different products; Figure S3: Motion trajectories of the Pt-SiO_2_ Janus micromotors in pure water within 12 s; Figure S4: The motion trajectories of Pt-SiO_2_ dimer micromotors in pure water within 12 s; Figure S5: Speed vs. time curves analyzed and plotted from Video S7; Video S1: Pt-SiO_2_ Janus micromotors in 10 wt% H_2_O_2_ solution; Video S2: Pt-SiO_2_ Janus micromotors in water; Video S3: Pt-SiO_2_ dimer micromotors in 10 wt% H_2_O_2_ solution; Video S4: Pt-SiO_2_ dimer micromotors in pure water; Video S5: Pt-SiO_2_ dimer micromotors in H_2_O_2_ solutions with different concentrations; Video S6: Pt-SiO_2_ micromotors, with transition structures between dimer and spherical Janus, in 10%wt H_2_O_2_ solution; Video S7: The hydroxyl-free (or FAS-17-modified) Pt-SiO_2_ dimers in water (from 0 to 4 s) and 10 wt% H_2_O_2_ solution (from 5 to 12 s).
