# Supplementary material for: Structurally Dependent Self-Propulsion Behaviors of Pt-SiO2 Micromotors
Source: Nanomaterials (Basel). 2026 Jan 4;16(1):73. doi: 10.3390/nano16010073 (PMC12787454; doi:10.3390/nano16010073)
Supplement: Supplementary file 1 [file nanomaterials-16-00073-s001.zip › nanomaterials-4051462-supplementary.pdf]

Supporting Information

Structurally Dependent Self-Propulsion Behaviors of Pt-SiO_2 Micromotors

Le Zhou [1,2,†], Qian Zhao [1,†], Hongwen Zhang [1,*], Haoming Bao [1] and Weiping Cai [1,*]

[1] Key Lab of Materials Physics, Anhui Key Lab of Nanomaterials and Nanotechnology, Institute of Solid State Physics, Hefei Institutes of Physical Science, Chinese Academy of Sciences, Hefei 230031, China; zhoule@aqnu.edu.cn (L.Z.); zhaoqian@issp.ac.cn (Q.Z.); baohm@issp.ac.cn (H.B.)

[2] Anhui Provincial Key Laboratory of Advanced Catalysis and Energy Materials, Anhui Ultra High Molecular Weight Polyethylene Fiber Engineering Research Center, School of Chemistry and Chemical Engineering, Anqing Normal University, Anqing 261433, China

* Correspondence: hwzhang@issp.ac.cn (H.Z.); wpcai@issp.ac.cn (W.C.); Tel.: +86-551-65592747 (W.C.); Fax: +86-551-65591434 (W.C.)

† These authors contributed equally to this work.

1. Preparation of hydroxyl-free-surfaced dimers

The hydroxyl-free Pt-SiO_2 dimers were prepared by decorating FAS-17 (1H,1H,2H,2H-perfluorodecyltrimethoxysilane) on the bare surface of the SiO_2 spheres, since silica can be made surface hydrophobic through modification with FAS-17 molecules. [34, 35] The as-prepared Pt-SiO_2 dimers were dispersed into 5 mL of xylene to form a suspension in a tube under ultrasonic vibration. After dropping 100 μL of FAS-17 into the suspension, the centrifuge tube was sealed and heated at 60 ^{o}C (water bath) for 24 h. The FAS-17-decorated Pt-SiO_2 dimers were thus obtained after cleaning and finally redispersed into 1 mL of water.

Video S1. Pt-SiO_2 Janus micromotors in 10 wt% H_2O_2 solution.

Video S2. Pt-SiO_2 Janus micromotors in water.

Video S3. Pt-SiO_2 dimer micromotors in 10 wt% H_2O_2 solution.

Video S4. Pt-SiO_2 dimer micromotors in pure water.

Video S5. Pt-SiO_2 dimer micromotors in H_2O_2 solutions with different concentrations: (a) 5 wt% and (b) 2.5 wt%.

Video S6. Pt-SiO_2 micromotors, with transition structures between dimer and spherical Janus, in 10%wt H_2O_2 solution: (a) Pt-SiO_2-150 (shown in Figure 3 a, b) and (b) Pt-SiO_2-90 (shown in Figure 3 c, d).

Video S7. The hydroxyl-free (or FAS-17-modified) Pt-SiO_2 dimers in water (from 0 to 4s) and 10 wt% H_2O_2 solution (from 5 to 12s).

Figure S1. **Le Zhou, et al.**

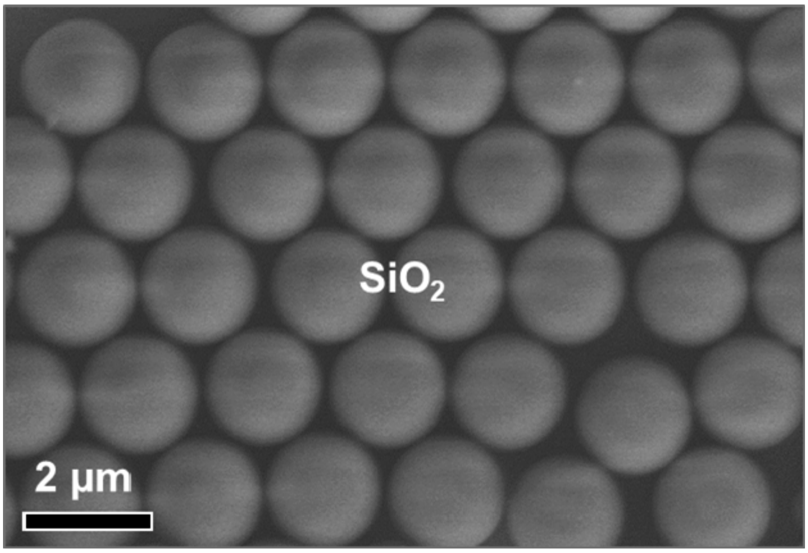

Figure S1. FESEM image of the obtained SiO_2 microsphere monolayer template.

Figure S2. Le Zhou, et al.

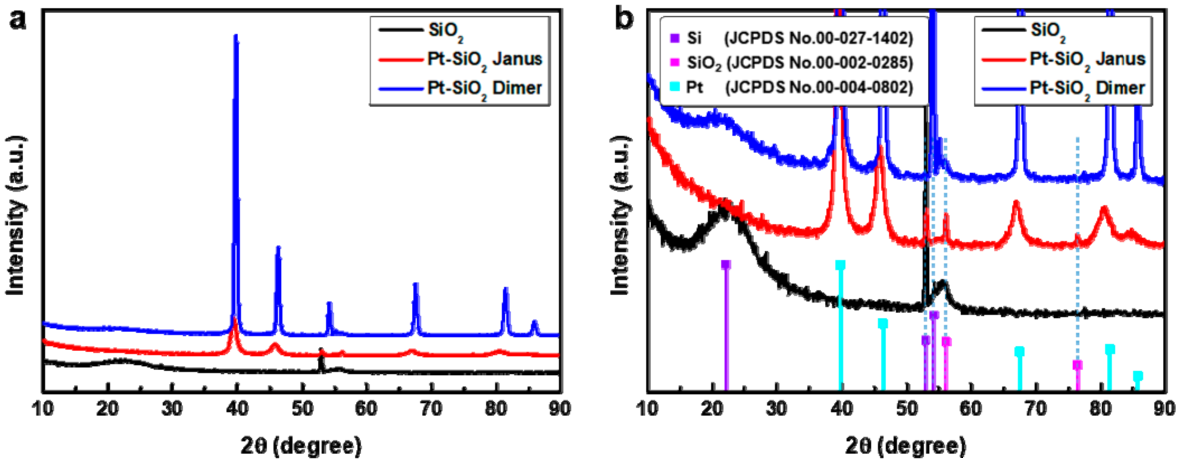

Figure S2. (a) XRD patterns of different products. The black, red, and blue curves correspond to the patterns of the SiO_2 microsphere monolayer template (Figure S1), the Pt-coated SiO_2 microsphere monolayer (Figure 1a or 1b) and the annealing products or Pt-SiO_2 dimer monolayer (Figure 1a or 1b) on Si wafer, respectively. (b) The enlarged plot of (a). The violet, magenta, and cyan line spectra correspond to the standard patterns of Si, SiO_2, and Pt, respectively.

Figure S3. Le Zhou, et al.

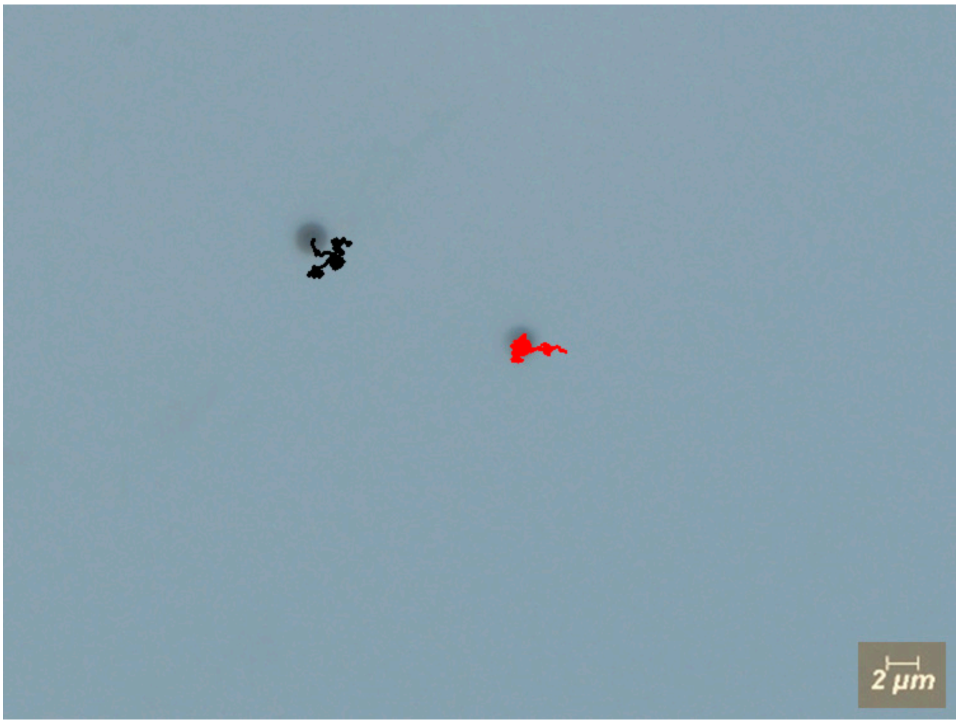

Figure S3. Motion trajectories of the Pt-SiO_2 Janus micromotors in pure water within 12 s.

Figure S4. Le Zhou, et al.

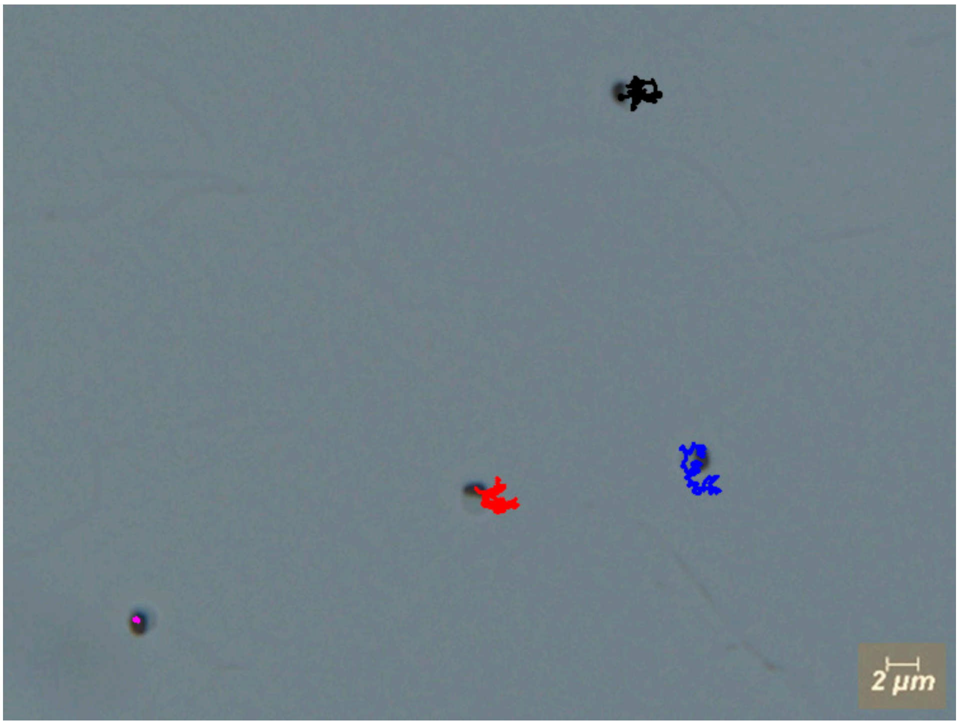

Figure S4. The motion trajectories of Pt-SiO_2 dimer micromotors in pure water within 12 s.

Figure S5. Le Zhou, et al.

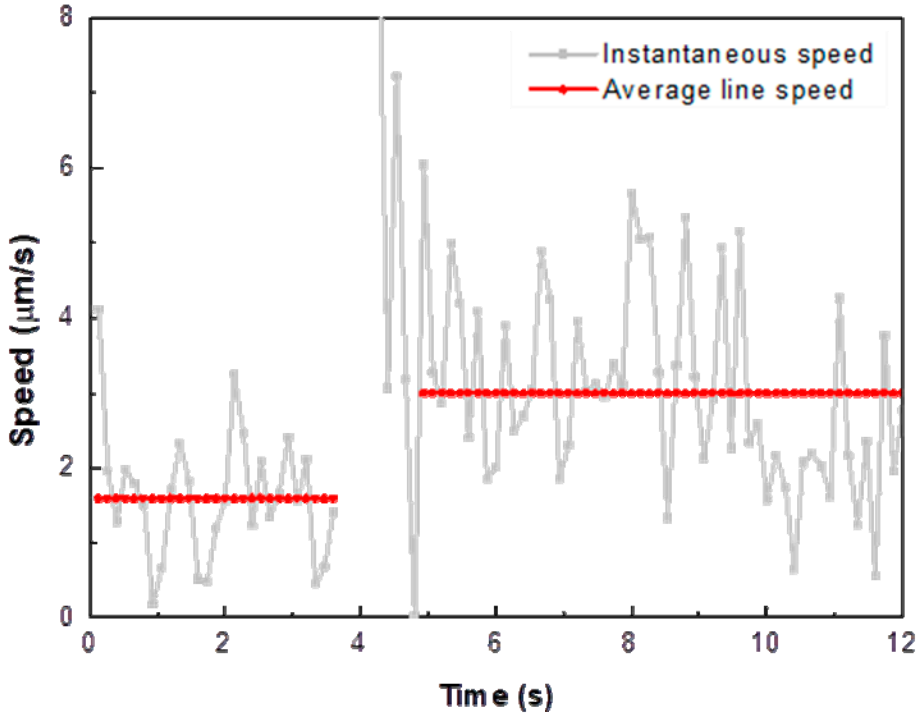

Figure S5. Speed vs. time curves analyzed and plotted from Video S7.
